# 2-(4-Morpholine­carbothio­ylsulfan­yl)­acetic acid

**DOI:** 10.1107/S1600536810012602

**Published:** 2010-04-14

**Authors:** Kong Mun Lo, Seik Weng Ng

**Affiliations:** a Department of Chemistry, University of Malaya, 50603 Kuala Lumpur, Malaysia

## Abstract

The asymmetric unit of the title compound, C_7_H_11_NO_3_S_2_, contains two independent mol­ecules with similar mol­ecular structures. The morpholine ring adopts a chair conformation, and the C_2_N—C(=S)—S fragment is planar in the two independent mol­ecules (r.m.s. deviations = 0.01 and 0.02 Å). The two mol­ecules are disposed about a false center of inversion and are held together by a pair of O—H⋯O hydrogen bonds. The crystal studied was a racemic twin; the minor twin component refined to 17%.

## Related literature

For the hydrogen-bonded dicyclo­hexyl­ammonium salt, see: Ng & Hook (1999 ▶). For the synthesis, see: Nachmias (1952 ▶).
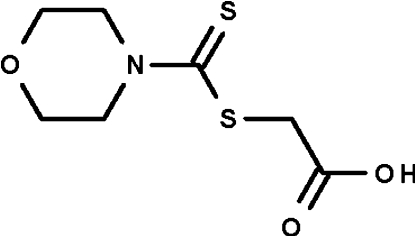


## Experimental

### 

#### Crystal data

C_7_H_11_NO_3_S_2_
*M* *_r_* = 221.29
Orthorhombic, 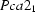

*a* = 14.7311 (3) Å
*b* = 4.7474 (1) Å
*c* = 28.0284 (5) Å
*V* = 1960.15 (7) Å^3^
*Z* = 8
Mo *K*α radiation
μ = 0.52 mm^−1^
*T* = 293 K
0.20 × 0.20 × 0.20 mm

#### Data collection

Bruker SMART APEX diffractometer
Absorption correction: multi-scan (*SADABS*; Sheldrick, 1996 ▶) *T*_min_ = 0.904, *T*_max_ = 0.904
16961 measured reflections
4495 independent reflections
3533 reflections with *I* > 2σ(*I*)
*R*_int_ = 0.060

#### Refinement

*R*[*F* ^2^ > 2σ(*F* ^2^)] = 0.066
*wR*(*F* ^2^) = 0.188
*S* = 1.11
4495 reflections
236 parameters
5 restraints
H-atom parameters constrained
Δρ_max_ = 1.52 e Å^−3^
Δρ_min_ = −0.37 e Å^−3^
Absolute structure: Flack (1983 ▶), 2199 Friedel pairs
Flack parameter: 0.2 (1)

### 

Data collection: *APEX2* (Bruker, 2009 ▶); cell refinement: *SAINT* (Bruker, 2009 ▶); data reduction: *SAINT*; program(s) used to solve structure: *SHELXS97* (Sheldrick, 2008 ▶); program(s) used to refine structure: *SHELXL97* (Sheldrick, 2008 ▶); molecular graphics: *X-SEED* (Barbour, 2001 ▶); software used to prepare material for publication: *pubCIF* (Westrip, 2010 ▶).

## Supplementary Material

Crystal structure: contains datablocks global, I. DOI: 10.1107/S1600536810012602/xu2747sup1.cif

Structure factors: contains datablocks I. DOI: 10.1107/S1600536810012602/xu2747Isup2.hkl

Additional supplementary materials:  crystallographic information; 3D view; checkCIF report

## Figures and Tables

**Table 1:** Hydrogen-bond geometry (Å, °)

*D*—H⋯*A*	*D*—H	H⋯*A*	*D*⋯*A*	*D*—H⋯*A*
O1—H1⋯O5	0.82	1.88	2.685 (7)	169
O4—H4⋯O2	0.82	1.88	2.689 (7)	170

**Fig. 1.**
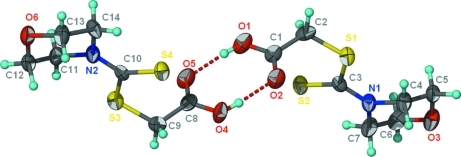
Thermal ellipsoid plot (Barbour, 2001) of the pair of C7H11NO3S2 molecules disposed about a false center-of-inversion at the 50% probability level. H atoms are drawn as spheres of arbitrary radius.

## supplementary crystallographic information

### Comment

The class of dithiocarbamyl-acetic acids, R_2_NC(S)SCH_2_CO_2_H, are synthetic plant growth-hormones. In an earlier study, the R_2_N = O(CH_2_CH_2_)_2_N derivative was characterized as the dicyclohexylammonium salt (Ng & Hook, 1999). The acid itself (Scheme I), exists as a hydrogen-bonded dimer, the two independent molecules being connected across a false center-of-inversion (Fig. 1, Table 1). The carboxyl –CO_2_ portions feature single as well as double carbon-oxygen bonds.

### Experimental

The carboxylic acid was synthesized from morpholine, carbon disulfide and chloroacetic acid (Nachmias, 1952), and was recrystallized from ethanol.

### Refinement

The carbon-carbon distances in the morpholine rings were retraied to 1.54±0.01 Å.

Hydrogen atoms were placed at calculated positions (C–H 0.97, O–H 0.82 Å) and were treated as riding on their parent atoms, with *U*(H) set to 1.2–1.5 times *U*_eq_(C,O). The final difference Fourier map had a peak 2.2 Å from S2.

The crystal is a racemic twin; the minor twin component refined to 17%.

### Crystal data

**Table:**

C_7_H_11_NO_3_S_2_	*F*(000) = 928
*M**_r_* = 221.29	*D*_x_ = 1.500 Mg m^−^^3^
Orthorhombic, *P**c**a*2_1_	Mo *K*α radiation, λ = 0.71073 Å
Hall symbol: P 2c -2ac	Cell parameters from 3880 reflections
*a* = 14.7311 (3) Å	θ = 2.9–25.7°
*b* = 4.7474 (1) Å	µ = 0.52 mm^−^^1^
*c* = 28.0284 (5) Å	*T* = 293 K
*V* = 1960.15 (7) Å^3^	Block, colorless
*Z* = 8	0.20 × 0.20 × 0.20 mm

### Data collection

**Table:**

Bruker SMART APEX diffractometer	4495 independent reflections
Radiation source: fine-focus sealed tube	3533 reflections with *I* > 2σ(*I*)
graphite	*R*_int_ = 0.060
ω scans	θ_max_ = 27.5°, θ_min_ = 1.5°
Absorption correction: multi-scan (*SADABS*; Sheldrick, 1996)	*h* = −19→19
*T*_min_ = 0.904, *T*_max_ = 0.904	*k* = −6→6
16961 measured reflections	*l* = −36→36

### Refinement

**Table:**

Refinement on *F*^2^	Secondary atom site location: difference Fourier map
Least-squares matrix: full	Hydrogen site location: inferred from neighbouring sites
*R*[*F*^2^ > 2σ(*F*^2^)] = 0.066	H-atom parameters constrained
*wR*(*F*^2^) = 0.188	*w* = 1/[σ^2^(*F*_o_^2^) + (0.1026*P*)^2^ + 0.7215*P*] where *P* = (*F*_o_^2^ + 2*F*_c_^2^)/3
*S* = 1.11	(Δ/σ)_max_ = 0.001
4495 reflections	Δρ_max_ = 1.52 e Å^−^^3^
236 parameters	Δρ_min_ = −0.36 e Å^−^^3^
5 restraints	Absolute structure: Flack (1983), 2199 Friedel pairs
Primary atom site location: structure-invariant direct methods	Flack parameter: 0.2 (1)

### Fractional atomic coordinates and isotropic or equivalent isotropic displacement parameters (Å^2^)

**Table:**

	*x*	*y*	*z*	*U*_iso_*/*U*_eq_	
S1	1.14478 (11)	0.9468 (4)	0.50019 (5)	0.0443 (3)	
S2	0.99909 (9)	0.7699 (3)	0.43159 (7)	0.0498 (3)	
S3	0.60753 (11)	0.5544 (4)	0.64749 (5)	0.0461 (4)	
S4	0.75227 (9)	0.7362 (3)	0.71543 (6)	0.0482 (3)	
O1	0.8902 (4)	1.0465 (12)	0.5349 (2)	0.0628 (15)	
H1	0.8469	0.9640	0.5468	0.094*	
O2	0.9876 (3)	0.7466 (9)	0.56506 (17)	0.0592 (12)	
O3	1.3067 (4)	0.5162 (10)	0.3578 (2)	0.0561 (15)	
O4	0.8628 (3)	0.4614 (10)	0.6158 (2)	0.0575 (14)	
H4	0.9054	0.5431	0.6030	0.086*	
O5	0.7633 (3)	0.7541 (9)	0.58260 (16)	0.0596 (12)	
O6	0.4379 (4)	0.9934 (10)	0.7892 (3)	0.0605 (16)	
N1	1.1727 (3)	0.6195 (11)	0.42631 (16)	0.0446 (11)	
N2	0.5791 (3)	0.8762 (10)	0.72174 (16)	0.0439 (10)	
C1	0.9717 (5)	0.9533 (15)	0.5416 (2)	0.0475 (16)	
C2	1.0451 (4)	1.1257 (11)	0.5176 (2)	0.0510 (13)	
H2A	1.0624	1.2764	0.5392	0.061*	
H2B	1.0190	1.2126	0.4895	0.061*	
C3	1.1053 (3)	0.7611 (10)	0.44898 (18)	0.0363 (10)	
C4	1.2672 (4)	0.6143 (15)	0.4401 (2)	0.0527 (14)	
H4A	1.2835	0.4266	0.4507	0.063*	
H4B	1.2770	0.7439	0.4663	0.063*	
C5	1.3265 (4)	0.6978 (12)	0.3981 (2)	0.0533 (14)	
H5A	1.3149	0.8925	0.3895	0.064*	
H5B	1.3900	0.6805	0.4068	0.064*	
C6	1.2189 (5)	0.5326 (16)	0.3440 (2)	0.055 (2)	
H6A	1.2094	0.4105	0.3168	0.066*	
H6B	1.2055	0.7241	0.3343	0.066*	
C7	1.1559 (5)	0.4494 (18)	0.3830 (3)	0.061 (2)	
H7A	1.0937	0.4761	0.3726	0.073*	
H7B	1.1642	0.2514	0.3903	0.073*	
C8	0.7808 (5)	0.5473 (14)	0.6087 (2)	0.0425 (14)	
C9	0.7074 (4)	0.3719 (11)	0.63020 (19)	0.0452 (12)	
H9A	0.7320	0.2777	0.6581	0.054*	
H9B	0.6906	0.2272	0.6074	0.054*	
C10	0.6437 (3)	0.7404 (10)	0.69901 (17)	0.0348 (10)	
C11	0.4816 (3)	0.8793 (13)	0.7084 (2)	0.0486 (13)	
H11A	0.4704	0.7443	0.6831	0.058*	
H11B	0.4646	1.0649	0.6970	0.058*	
C12	0.4262 (4)	0.8035 (13)	0.7521 (2)	0.0528 (14)	
H12A	0.3625	0.7975	0.7434	0.063*	
H12B	0.4436	0.6170	0.7628	0.063*	
C13	0.5344 (5)	0.9823 (16)	0.8041 (3)	0.056 (2)	
H13A	0.5481	0.7961	0.8163	0.067*	
H13B	0.5445	1.1172	0.8295	0.067*	
C14	0.5974 (4)	1.0483 (14)	0.7627 (2)	0.0462 (15)	
H14A	0.5907	1.2449	0.7540	0.055*	
H14B	0.6597	1.0191	0.7727	0.055*	

### Atomic displacement parameters (Å^2^)

**Table:**

	*U*^11^	*U*^22^	*U*^33^	*U*^12^	*U*^13^	*U*^23^
S1	0.0386 (8)	0.0618 (7)	0.0325 (7)	−0.0035 (7)	−0.0021 (6)	−0.0066 (9)
S2	0.0345 (6)	0.0625 (9)	0.0525 (7)	−0.0025 (6)	−0.0064 (5)	−0.0034 (6)
S3	0.0434 (8)	0.0601 (7)	0.0348 (7)	−0.0063 (7)	−0.0038 (6)	−0.0045 (9)
S4	0.0357 (6)	0.0615 (8)	0.0476 (6)	−0.0024 (6)	−0.0059 (6)	0.0009 (6)
O1	0.061 (3)	0.071 (3)	0.056 (3)	0.023 (3)	0.011 (3)	0.006 (2)
O2	0.055 (3)	0.065 (3)	0.058 (2)	0.013 (2)	0.009 (2)	0.020 (2)
O3	0.047 (3)	0.083 (4)	0.039 (3)	0.013 (2)	0.013 (3)	−0.0063 (18)
O4	0.044 (3)	0.069 (3)	0.059 (3)	0.019 (2)	0.013 (2)	0.028 (2)
O5	0.061 (3)	0.060 (3)	0.058 (3)	0.020 (2)	0.012 (2)	0.028 (2)
O6	0.034 (3)	0.069 (4)	0.078 (4)	0.0018 (17)	0.000 (3)	−0.011 (2)
N1	0.032 (2)	0.061 (3)	0.041 (2)	−0.003 (2)	−0.0004 (18)	−0.011 (2)
N2	0.032 (2)	0.056 (3)	0.043 (3)	−0.002 (2)	−0.0055 (18)	−0.010 (2)
C1	0.054 (4)	0.050 (3)	0.039 (3)	0.012 (3)	0.003 (3)	0.013 (3)
C2	0.073 (4)	0.039 (3)	0.041 (3)	0.011 (3)	0.004 (3)	−0.006 (2)
C3	0.037 (3)	0.040 (3)	0.032 (2)	−0.0069 (19)	−0.0011 (17)	0.003 (2)
C4	0.042 (3)	0.069 (4)	0.048 (3)	0.002 (3)	−0.003 (2)	−0.004 (3)
C5	0.036 (3)	0.051 (3)	0.073 (4)	0.004 (2)	0.006 (3)	0.008 (3)
C6	0.064 (5)	0.069 (4)	0.033 (4)	0.011 (3)	−0.002 (3)	−0.012 (3)
C7	0.044 (4)	0.073 (4)	0.066 (5)	0.004 (3)	−0.005 (3)	−0.036 (4)
C8	0.050 (4)	0.049 (3)	0.028 (3)	0.005 (3)	0.006 (2)	−0.009 (2)
C9	0.056 (3)	0.040 (3)	0.039 (3)	−0.005 (3)	0.005 (2)	0.001 (2)
C10	0.036 (2)	0.040 (3)	0.029 (2)	−0.0089 (19)	−0.0042 (18)	0.007 (2)
C11	0.030 (2)	0.062 (4)	0.053 (3)	0.006 (3)	−0.011 (2)	−0.006 (3)
C12	0.031 (3)	0.053 (3)	0.074 (4)	−0.003 (2)	−0.003 (2)	0.003 (3)
C13	0.037 (4)	0.080 (5)	0.051 (5)	0.003 (3)	0.000 (3)	−0.005 (3)
C14	0.044 (3)	0.058 (3)	0.037 (3)	−0.015 (3)	0.007 (3)	−0.009 (3)

### Geometric parameters (Å, °)

**Table:**

S1—C2	1.765 (6)	C2—H2B	0.9700
S1—C3	1.782 (5)	C4—C5	1.518 (7)
S2—C3	1.640 (5)	C4—H4A	0.9700
S3—C10	1.774 (5)	C4—H4B	0.9700
S3—C9	1.775 (6)	C5—H5A	0.9700
S4—C10	1.664 (5)	C5—H5B	0.9700
O1—C1	1.294 (8)	C6—C7	1.487 (8)
O1—H1	0.8200	C6—H6A	0.9700
O2—C1	1.204 (8)	C6—H6B	0.9700
O3—C6	1.352 (10)	C7—H7A	0.9700
O3—C5	1.450 (9)	C7—H7B	0.9700
O4—C8	1.291 (8)	C8—C9	1.492 (9)
O4—H4	0.8200	C9—H9A	0.9700
O5—C8	1.251 (8)	C9—H9B	0.9700
O6—C12	1.387 (8)	C11—C12	1.514 (7)
O6—C13	1.481 (10)	C11—H11A	0.9700
N1—C3	1.357 (7)	C11—H11B	0.9700
N1—C4	1.444 (7)	C12—H12A	0.9700
N1—C7	1.479 (8)	C12—H12B	0.9700
N2—C10	1.314 (7)	C13—C14	1.519 (7)
N2—C14	1.434 (8)	C13—H13A	0.9700
N2—C11	1.484 (6)	C13—H13B	0.9700
C1—C2	1.514 (9)	C14—H14A	0.9700
C2—H2A	0.9700	C14—H14B	0.9700
			
C2—S1—C3	100.9 (3)	N1—C7—C6	110.7 (6)
C10—S3—C9	102.5 (3)	N1—C7—H7A	109.5
C1—O1—H1	120.0	C6—C7—H7A	109.5
C6—O3—C5	112.3 (5)	N1—C7—H7B	109.5
C8—O4—H4	120.0	C6—C7—H7B	109.5
C12—O6—C13	107.9 (5)	H7A—C7—H7B	108.1
C3—N1—C4	126.0 (4)	O5—C8—O4	122.1 (6)
C3—N1—C7	122.2 (5)	O5—C8—C9	121.7 (6)
C4—N1—C7	111.8 (5)	O4—C8—C9	116.1 (6)
C10—N2—C14	122.1 (4)	C8—C9—S3	116.0 (4)
C10—N2—C11	125.7 (4)	C8—C9—H9A	108.3
C14—N2—C11	112.2 (5)	S3—C9—H9A	108.3
O2—C1—O1	122.6 (7)	C8—C9—H9B	108.3
O2—C1—C2	123.0 (6)	S3—C9—H9B	108.3
O1—C1—C2	114.4 (6)	H9A—C9—H9B	107.4
C1—C2—S1	117.2 (4)	N2—C10—S4	124.6 (4)
C1—C2—H2A	108.0	N2—C10—S3	114.9 (4)
S1—C2—H2A	108.0	S4—C10—S3	120.5 (3)
C1—C2—H2B	108.0	N2—C11—C12	108.4 (4)
S1—C2—H2B	108.0	N2—C11—H11A	110.0
H2A—C2—H2B	107.2	C12—C11—H11A	110.0
N1—C3—S2	124.9 (4)	N2—C11—H11B	110.0
N1—C3—S1	112.6 (4)	C12—C11—H11B	110.0
S2—C3—S1	122.6 (3)	H11A—C11—H11B	108.4
N1—C4—C5	110.1 (5)	O6—C12—C11	112.6 (5)
N1—C4—H4A	109.6	O6—C12—H12A	109.1
C5—C4—H4A	109.6	C11—C12—H12A	109.1
N1—C4—H4B	109.6	O6—C12—H12B	109.1
C5—C4—H4B	109.6	C11—C12—H12B	109.1
H4A—C4—H4B	108.2	H12A—C12—H12B	107.8
O3—C5—C4	109.4 (5)	O6—C13—C14	111.3 (7)
O3—C5—H5A	109.8	O6—C13—H13A	109.4
C4—C5—H5A	109.8	C14—C13—H13A	109.4
O3—C5—H5B	109.8	O6—C13—H13B	109.4
C4—C5—H5B	109.8	C14—C13—H13B	109.4
H5A—C5—H5B	108.2	H13A—C13—H13B	108.0
O3—C6—C7	111.9 (6)	N2—C14—C13	112.3 (5)
O3—C6—H6A	109.2	N2—C14—H14A	109.2
C7—C6—H6A	109.2	C13—C14—H14A	109.2
O3—C6—H6B	109.2	N2—C14—H14B	109.2
C7—C6—H6B	109.2	C13—C14—H14B	109.1
H6A—C6—H6B	107.9	H14A—C14—H14B	107.9
			
O2—C1—C2—S1	29.1 (9)	O5—C8—C9—S3	−35.2 (8)
O1—C1—C2—S1	−150.9 (6)	O4—C8—C9—S3	149.5 (6)
C3—S1—C2—C1	74.8 (5)	C10—S3—C9—C8	−72.7 (5)
C4—N1—C3—S2	178.2 (5)	C14—N2—C10—S4	2.9 (7)
C7—N1—C3—S2	−1.4 (8)	C11—N2—C10—S4	−178.8 (4)
C4—N1—C3—S1	−0.7 (7)	C14—N2—C10—S3	−176.9 (4)
C7—N1—C3—S1	179.8 (5)	C11—N2—C10—S3	1.4 (7)
C2—S1—C3—N1	175.4 (4)	C9—S3—C10—N2	−174.2 (4)
C2—S1—C3—S2	−3.5 (4)	C9—S3—C10—S4	6.0 (4)
C3—N1—C4—C5	−126.6 (6)	C10—N2—C11—C12	128.6 (5)
C7—N1—C4—C5	53.0 (8)	C14—N2—C11—C12	−53.0 (7)
C6—O3—C5—C4	59.7 (7)	C13—O6—C12—C11	−63.0 (7)
N1—C4—C5—O3	−55.2 (7)	N2—C11—C12—O6	61.0 (7)
C5—O3—C6—C7	−60.0 (8)	C12—O6—C13—C14	57.6 (7)
C3—N1—C7—C6	127.4 (7)	C10—N2—C14—C13	−130.6 (6)
C4—N1—C7—C6	−52.2 (9)	C11—N2—C14—C13	50.9 (8)
O3—C6—C7—N1	55.3 (8)	O6—C13—C14—N2	−52.6 (8)

### Hydrogen-bond geometry (Å, °)

**Table:**

*D*—H···*A*	*D*—H	H···*A*	*D*···*A*	*D*—H···*A*
O1—H1···O5	0.82	1.88	2.685 (7)	169
O4—H4···O2	0.82	1.88	2.689 (7)	170
